# Mitochondrial Dysfunction in Neural Injury

**DOI:** 10.3389/fnins.2019.00030

**Published:** 2019-02-04

**Authors:** Xiu-Yun Zhao, Mei-Hong Lu, De-Juan Yuan, De-En Xu, Pei-Pei Yao, Wen-Li Ji, Hong Chen, Wen-Long Liu, Chen-Xiao Yan, Yi-Yuan Xia, Shao Li, Jin Tao, Quan-Hong Ma

**Affiliations:** ^1^Institute of Neuroscience and Jiangsu Key Laboratory of Neuropsychiatric Diseases, Soochow University, Suzhou, China; ^2^Department of Neurology and Suzhou Clinical Research Center of Neurological Disease, The Second Affiliated Hospital of Soochow University, Suzhou, China; ^3^Department of Physiology, Liaoning Provincial Key Laboratory of Cerebral Diseases, Dalian Medical University, Dalian, China; ^4^Wuxi No. 2 People’s Hospital, Wuxi, China; ^5^Department of Physiology and Neurobiology and Centre for Ion Channelopathy, Medical College of Soochow University, Suzhou, China

**Keywords:** mitochondrial dysfunction, neural injury, mitochondria, neurodegeneration, neurological disorders

## Abstract

Mitochondria are the double membrane organelles providing most of the energy for cells. In addition, mitochondria also play essential roles in various cellular biological processes such as calcium signaling, apoptosis, ROS generation, cell growth, and cell cycle. Mitochondrial dysfunction is observed in various neurological disorders which harbor acute and chronic neural injury such as neurodegenerative diseases and ischemia, hypoxia-induced brain injury. In this review, we describe how mitochondrial dysfunction contributes to the pathogenesis of neurological disorders which manifest chronic or acute neural injury.

## Introduction

Mitochondria structurally consist of two membranes (the inner and outer membrane), an intermembrane space and an internal matrix which contains mtDNA. Mitochondria are known as the “power house,” which provide 95% energy for cells. Mitochondria are important for calcium signaling (Brini et al., 2014), cell apoptosis (Tait and Green, 2013), production and sequestration of ROS (Shadel and Horvath, 2015). Mitochondrial dysfunction causes impairment in these processes such as impaired energy supply, Ca^2+^ buffering, increased ROS production and enhanced apoptosis, which contribute to neurodegeneration. In addition, mitochondria modulate synaptic plasticity via regulating neurotransmitter production and inactivation, the formation and maintenance of synapses, neuronal development, neurogenesis, axonal transport, synaptic plasticity (Levy et al., 2003; Cheng et al., 2010, 2012; Lopez-Domenech et al., 2016; Todorova and Blokland, 2017), which are closely linked to neurological disorders.

Mitochondria are dynamic organelles that adapt to physiological needs in different tissues. Neurons are dependent on mitochondrial oxidative phosphorylation (OXPHO) to fulfill their energy demands. Neurons have a limited capacity to upregulate glycolysis. In comparison to neurons, astrocytes, and oligodendrocytes are highly glycolytic, and are resilient to mitochondrial dysfunction (Dimonte et al., 1992; Fernandez-Fernandez et al., 2012). The number and morphology of mitochondria vary among cell types. Even in the same type cells, mitochondria change their number and morphology in response to different environments. Mitochondria keep a balance in their number, structure and function (mitochondrial homeostasis) (Van Blerkom, 2009), which plays an important physiological role in maintaining cell homeostasis (Fischer et al., 2012; Marzetti et al., 2013; Muller et al., 2015). Impairment in mitochondrial homeostasis was observed in neurological disorders which manifest chronic or acute neural injury such as neurodegenerative diseases, cerebral ischemia, cerebral hypoxia and other brain injuries (Calkins et al., 2011; Witte et al., 2014; Liu et al., 2016; Suarez-Rivero et al., 2016; Ottolini et al., 2017; Cheng and Bai, 2018; Ludwig et al., 2018).

## Mitochondrial Homeostasis

The morphology and function of mitochondrial networks are regulated by continuous fusion and fission cycles, which constitutes a quality control system to maintain mitochondrial function. When the mitochondria are damaged, the damaged mitochondria fuse with the surrounding healthy mitochondria, thus alleviating the slight damage. If the mitochondria are severely damaged, the damaged mitochondria will be transported to the lysosomes for degradation through a process called mitophagy. While new mitochondria continue to divide to maintain the number of qualified mitochondria. By these methods, the cells control dynamic balance of the mitochondrial network, thus maintaining cell homeostasis (Fischer et al., 2012; Marzetti et al., 2013; Muller et al., 2015).

### Mitochondrial Fission and Fusion

Mitochondrial fission/fusion refers to the mutual fusion and recleavage of two lipid bilayers of mitochondria (Hoppins et al., 2007). Mitochondrial fission and fusion play critical roles in maintaining mitochondrial homeostasis when cells experience metabolic or environmental stresses. When the mitochondria are damaged, fusion rescues stress by allowing functional mitochondria to complement dysfunctional mitochondria by diffusion and sharing of components between organelles. Fission is needed to create new mitochondria. However, it also contributes to the mitochondrial quality control by enabling the removal of damaged mitochondria (Youle and van der Bliek, 2012; Meyer et al., 2017).

Fission is mediated by members of the cytoplasmic dynein family [Dynamin1 (Dnm1) in yeast, worms, and Dynamin-related protein1 (Drp1) in flies and mammals]. Drp1 is recruited from the cytoplasm to spiral around the mitochondria, contracting to sever internal and external membranes. Yeast shares the common function of Drp1 with mammals but requiring a unique accessory protein. Mitochondrial division protein 1 (Mdv1) recruits Dnm1 to the mitochondrial fission site in yeast. Whereas mitochondrial dynamics protein 49 (Mid49), Mid51, and mitochondrial fission factor (Mff) recruit Drp1 to sites of mitochondrial and endoplasmic reticulum contact in mammals (Friedman et al., 2011; Elgass et al., 2013). Fusion between mitochondrial outer membranes is mediated by mitofusin 1 (Mfn1) and Mfn2, members of the membrane anchoring motor protein family in mammals. Whereas fusion between mitochondrial inner membranes is mediated by a single dynamin family member, Opal (Dynamin-like 120 kDa protein, mitochondrial 1, in mammals). Mitochondrial fission and fusion mechanisms are regulated by protein levels and post-translational modifications (Hoppins et al., 2007).

### Mitochondrial Transport

A variety of studies have shown that mitochondrial transport is impaired in patients with neurodegenerative diseases or brain injury. Mitochondrial transport in neuronal cells is closely related to neurological diseases (Ebrahimi-Fakhari et al., 2016). Mitochondria are a power factory for cells that supply a large amount of ATP to meet the physiological needs of neurons. However, due to the complex structure of neurons, it is difficult to compare the energy requirements with ordinary cells (Zhou et al., 2014). Compared to ordinary cells, neurons need to transport mitochondria to distal synapses and therefore have higher energy requirements. The process of mitochondrial transport from the cell body to the axon is anterograde transport, and the process of transporting from the axon back to the cell body is the retrograde transport. The anterograde transport of mitochondria in normal neurons maintains a dynamic balance with the retrograde transport. When the neurons are induced by external pressure or the integrity is impaired, the damaged mitochondria preferentially degenerate from the axon transported cysts (Cai et al., 2012). The balance with retrograde transportation is broken.

### Mitophagy

Mitophagy refers to the process of cells selectively removing excess or damaged mitochondria through autophagy, which plays an important role in the mitochondrial quality control and cell survival (Pickles et al., 2018). There are many ways to regulate mitophagy. The PINK1/Parkin pathway has emerged as a critical pathway in the regulation of mitophagy as well as mitochondrial function (Clark et al., 2006; Narendra et al., 2008). Dysfunctional mitochondria fail to import and degrade PINK1, which stabilizes on the outer mitochondrial membrane (OMM). After PINK1 accumulation, PINK1 phosphorylates ubiquitin and Parkin to activate the E3 ligase activity of Parkin. Parkin ubiquitinates substrates on the outer mitochondria for two divergent processes: autophagosome recruitment and ubiquitin-proteasome degradation of ubiquitinated mitochondrial substrates. Then autophagy receptors such as NBR1, Nix, TAxBP1, NDP51, optineurin and FUNDC1 on the outer membrane binds LC3 to recruit the autophagosomes around the damaged mitochondria. The autophagosome is then delivered to the lysosome for degradation (Pickrell and Youle, 2015; Chu, 2018). If mitochondria are damaged irreversibly, they will initiate autophagic clearance. Impaired mitophagy is closely related to brain injury in ischemic stroke and neurodegenerative diseases.

## Mitochondrial Dysfunction in Hypoxic-Ischemic Brain Injury

**FIGURE 1:**
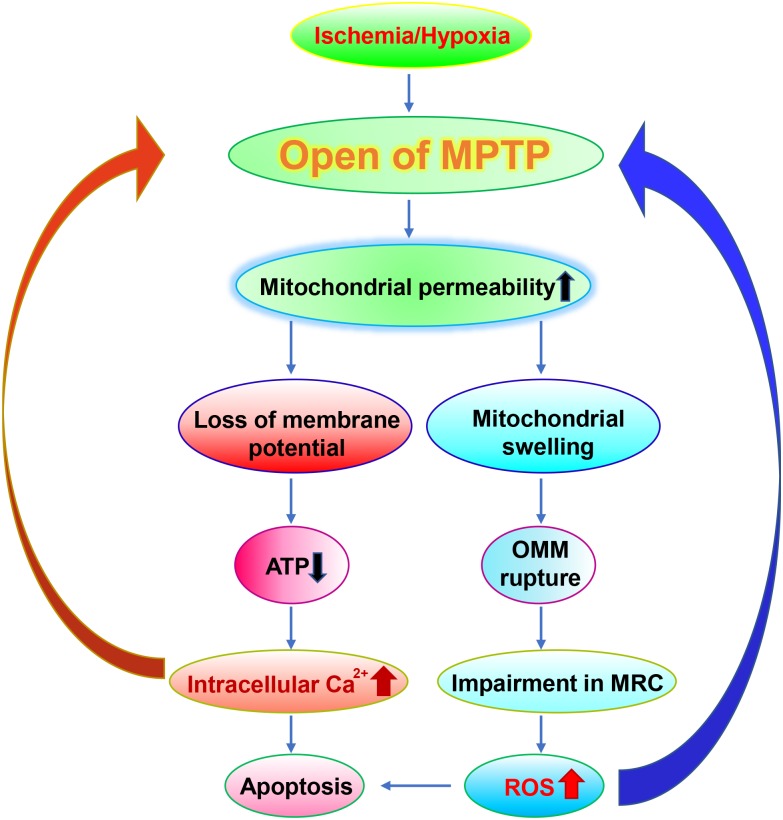
Roles of MPTP in ischemia. In the ischemia, MPTP is open, which leads to loss of the membrane potential and the rupture of OMM. The damaged mitochondria exhibit reduced ATP generation, impaired MRC and increased Ca^2+^ flux which results in elevated generation of ROS and apoptosis respectively. It is worth noting that elevated ROS levels and apoptosis in turn further exacerbate the damages on MPTP.

Mitochondria are composed of two membrane structures with high permeability of the outer membrane and relatively low permeability of the endometrium. The mitochondrial permeability transition pore (MPTP) is a non-specific voltage-dependent special protein complex which crosses the mitochondrial outer membrane and control the mitochondrial permeability (Halestrap, 2009). In the physiological state, MPTP is in the off state. Whereas MPTP is open during ischemia, which is triggered by both the Ca^2+^ overload in the mitochondrial matrix and the elevated oxidative stress (Malkevitch et al., 1997; Kushnareva and Sokolove, 2000; Kim et al., 2006). The opening of MPTP leads to the increase of mitochondrial permeability, which allows the solute such as water, large molecules and ions to enter freely into the mitochondrial matrix. The latter in turn causes mitochondrial swelling, the outer membrane to rupture, the impairment of the MRC, leading to an abundant release of reactive oxygen species (ROS) (Broekemeier et al., 1998; Krasnikov et al., 2005). Moreover, the increased mitochondrial permeability also causes loss of membrane potential, further leading to decreased cellular mitochondrial ATP levels and enhanced intracellular Ca^2+^ concentration (Zorov et al., 2000), and then activation of endogenous apoptotic pathways, consequently inducing ischemia-hypoxia-induced neuronal damage (Broughton et al., 2009) (Figure 1). Consistent with these observations, the immunosuppressant compound cyclosporine A, which is capable of inhibiting MPTP, exhibits a neuroprotective effect on acute brain injury caused by ischemia (Sullivan et al., 2000; Uchino, 2002) (Table 1).

**Table 1:** Compounds targeting mitochondria in the treatment of ischemia, PD, and AD.

Compounds	Mechanisms of action	Disorders	Therapeutic effects	Reference
Cyclosporine A	Inhibiting MPTP	Ischemia-caused acute brain injury	Pre-clinical studies	Sullivan et al., 2000; Uchino, 2002
Tirilazad mesylate	Lipid peroxidation inhibitor	Ischemia-caused brain injury	Effective in pre-clinical models; showing no effective in clinical studies	Park and Hall, 1994; Fleishaker et al., 1995
Edaravone	Free radical scavenger	Ischemia-caused brain injury	Exhibited clinical improvements in acute ischemic stroke patients	Edaravone Acute Infarction Study Group, 2003
Lubeluzole	Reduces NO levels and subsequent ONOO^-^ production	Ischemia-caused brain injury	Effective in pre-clinical models; showing no effective in clinical studies	De Ryck et al., 1996; Diener et al., 1996
DPQ	Inhibiting PARP-1	Ischemia-caused brain injury	Pre-clinical studies; partial protection against the pathological process	Soane et al., 2007
Mdivi-1	Inhibitor of Drp-1	Ischemia-caused brain injury	Pre-clinical studies; controversial effect	Baek et al., 2017
3-MA	Inhibiting autophagy/mitophagy	Ischemia-caused brain injury	Pre-clinical studies; controversial effect	Zhang X. et al., 2013; Zuo et al., 2014
Rapamycin	Autophagy/mitophgy inducer through suppressing mTOR	Ischemia-caused brain injury	Pre-clinical studies	Li et al., 2014
Selegiline	Inhibiting monoamine oxidase-B (MAO-B) and antioxidant	Parkinson’s disease	Beneficial effect and significantly delaying the time of using levodopa	Koller et al., 1993
Rasagiline	MAO-B inhibitor	Parkinson’s disease	Change the Unified Parkinson Disease Rating Scale (UPDRS) modestly	Smith et al., 2015
*N*-acetylcysteine (NAC)	Antioxidant	Parkinson’s disease	Improved UPDRS moderately	Monti et al., 2016
Mitoquinone	Mitochondrial targeted antioxidant	Parkinson’s disease	Protected dopaminergic neurons in a 6-OHDA-induced model of PD; but no effective in clinical studies	Snow et al., 2010; Liu and Wang, 2014; Xi et al., 2018
Ubiquinone	Antioxidant	Parkinson’s disease	Pre-clinical studies	Koller et al., 1993
Creatine	Antioxidant	Parkinson’s disease	Pre-clinical studies	Kieburtz et al., 2015
Curcumin	Antioxidant	Alzheimer’s disease	Pre-clinical studies	Mecocci and Polidori, 2012; Zhao and Zhao, 2013
Mitoquinone	Mitochondria-targeted antioxidant	Alzheimer’s disease	Pre-clinical studies	Mecocci and Polidori, 2012; Zhao and Zhao, 2013
CoQ10/Ubisol-Q10	Antioxidants	Alzheimer’s disease	Pre-clinical studies	McManus et al., 2011; Muthukumaran et al., 2018
Idebenone	An analog of CoQ10	Alzheimer’s disease	Effective in clinical trails	Senin et al., 1992; Weyer et al., 1997
Nicotinamide riboside (NR)	NAD^+^ precursors	Alzheimer’s disease	Pre-clinical studies	Wang et al., 2016; Hou et al., 2018
Nicotinamide mononucleotide (NMN)	NAD^+^ precursors	Alzheimer’s disease	Pre-clinical studies	Wang et al., 2016; Hou et al., 2018
Rapamycin	Autophagy/mitophgy inducer through suppressing mTOR	Alzheimer’s disease	Pre-clinical studies	Spilman et al., 2010; Caccamo et al., 2013
Trehalose	mTOR-independent autophagy/mitophgy inducer	Alzheimer’s disease	Pre-clinical studies	Spilman et al., 2010; Caccamo et al., 2013
2, 4- Dinitrophenol (DNP)	Mitochondrial uncoupling agents	Alzheimer’s disease	Pre-clinical studies	Geisler et al., 2017


**FIGURE 2:**
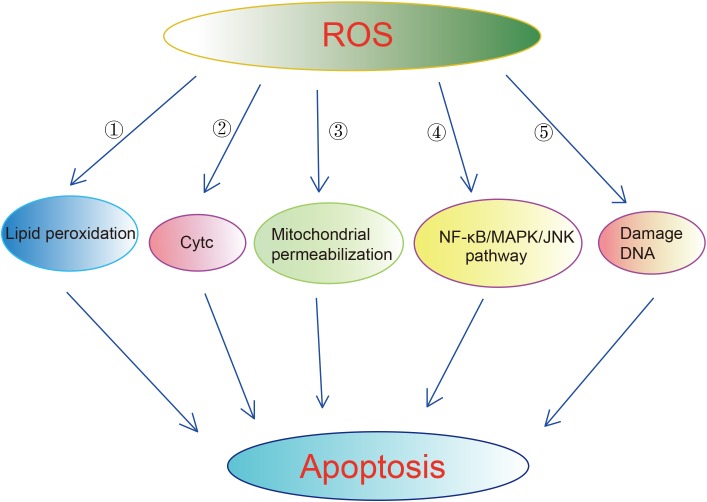
Mechanisms underlying that ROS induced apoptosis. Excessive ROS can cause apoptosis via multiple ways: 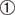 enhancing lipid peroxidation. 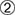 Releasing of Cytc. 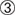 Enhancing mitochondrial permeabilization. 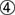 Activation of NF-KB/MAPK/JNK pathway. 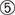 Damaging DNA directly.

Mitochondria and its electron transport chain complexes are the main sources of ROS. Approximately 90% of cellular ROS production occurs in the mitochondria. Healthy mitochondria generate small levels of ROS. There is a set of scavenging system including enzymatic and non-enzymatic antioxidants to protect cells from the attack by ROS. The enzymatic antioxidants include superoxide dismutase (SOD), glutathione peroxidase (GPx), and catalase (CAT). The non-enzymatic antioxidants include ascorbic acid (Vitamin C), α-tocopherol (Vitamin E), glutathione (GSH), carotenoids, flavonoids, and other antioxidants (Valko et al., 2007). Under normal circumstances, ROS exhibits little damage as it enters into a balance with antioxidant systems (Fridovich, 1995; Takizawa et al., 1998). However, excessive ROS damages proteins, mtDNA and lipids, resulting in apoptosis, neuroinflammation, and disruption of blood–brain barrier (BBB) in ischemic brains (Shirley et al., 2014). Excessive ROS induces apoptosis through lipid peroxidation. 4-Hydroxynoneal (4-HNE), one of byproducts of lipid peroxidation, has been shown to be upregulated in rat brains upon cerebral ischemia and to induce axonal damage and apoptosis of oligodendrocytes (McCracken et al., 2000; Matsuda et al., 2009). ROS can also lead to apoptosis through releasing of cytochrome C (CytC) (Kirkland et al., 2002; Sugawara et al., 2002), enhancing mitochondrial permeabilization (Kim et al., 2006), activation of NF-κB/MAPK/JNK pathway (Kim et al., 2010). Excessive ROS also can bring damages directly on DNA, leading to DNA base damage and single-strand breaks (SSBs) in rodent models of cerebral ischemia (Liu et al., 1996; Chen et al., 1997) (Figure 2). Excessive ROS results in the breakdown of BBB through direct activation of matrix metalloproteinase (MMPs) and regulating Ca^2+^ concentration (Mark and Davis, 2002; Yamagata et al., 2004; Cortez et al., 2007; Rosell et al., 2008). The generation of ROS is elevated in the ischemic brains. In ischemia, oxygen is deleted prior to glucose, which results in an accumulation of lactic acid. The latter promotes pro-oxidant and detrimental changes in neurons such as the inactivation of antioxidant defenses, release of oxidant ions and elevated glutamate toxicity (Manzanero et al., 2013). In addition, the enzyme xanthin oxidase and mitochondrial depolarization also contributes to the elevated ROS in the ischemic brains (Abramov et al., 2007). It is worth noting that blood reperfusion also leads to a substantial increase in mitochondrial ROS, which may be contributed by the complex I of MRC (Niatsetskaya et al., 2012). In this context, it is reported that ischemic stroke upregulates the antioxidant defense system mentioned above (Fridovich, 1995; Takizawa et al., 1998). In contrast, some studies detected reduced levels of antioxidants in stroke patients in the early hours of post-stroke (Kaur et al., 2011). Despite these controversial reports, it is believed that the antioxidant defense system fails to protect cells from the elevated ROS, eventually leading to cell death (Manzanero et al., 2013). Considering the roles of ROS in ischemia, anti-oxidants have been used to treat ischemia. One of the anti-oxidant strategies is using compounds capable of scavenging free radicals such as tirilazad mesylate, an inhibitor of lipid peroxidation. Tirilazad exhibited benefits in reducing infarct size in pre-clinical models (Xue et al., 1992; Park and Hall, 1994). However, tirilazad failed to improve mortality in clinical studies, which possibly due to differences metabolic rate between women and men (Fleishaker et al., 1995; Scott et al., 1996). Another free radical scavenger, edaravone, which has been widely used in clinics in Japan for the treatment of cerebral infarctions, exhibited clinical improvements in acute ischemic stroke patients (Edaravone Acute Infarction Study Group, 2003).

In addition to ROS, mitochondrial dysfunction also leads to abnormal generation of nitrix oxide (NO), which in turn regulates mitochondrial function. One of mechanisms underlying that NO-regulating mitochondrial function is via S-nitrosylation of mitochondrial proteins. About 1% mitochondrial proteins are available for S-nitrosylation. NO inhibits Complex I (Clementi et al., 1998; Dahm et al., 2006) and Complex IV (Cleeter et al., 1994; Orsi et al., 2000) and other metabolic enzymes such as mitochondrial aldehyde dehydrogenase (ALDH2) (Moon et al., 2005) and aconitase (Tortora et al., 2007) via S-nitrosylation. S-nitrosylation is also involved in regulation of MPTP, which is closely linked to neural injury in stroke (Vieira and Kroemer, 2003). Moreover, S-nitrosylated SHP-2 and receptor-interacting protein 3 (RIP3) contributes to NMDA receptor-mediated excitotoxicity in acute ischemic stroke (Shi et al., 2013; Miao et al., 2015). Consistently, mitochondria-targeted S-nitrosothiols (MitoSNOs), which generates NO and S-nitrosated thiol proteins within the mitochondria, has been observed protective in mouse model of cardiac ischemia-refusion injury (Prime et al., 2009). Lubeluzole, which reduces NO levels and subsequent ONOO- production, reduced infarct in a transient MCAO mouse model (Aronowski et al., 1996; De Ryck et al., 1996). However, lubeluzole showed no improvement in mortality in a large clinical study despite the fact that it shows benefits in two smaller clinical studies (Diener et al., 1996; Diener, 1998; Grotta, 1998) (Table 1).

A great amount of cellular NAD(H) is involved in mitochondria, the ratio of NAD/NADH plays an important role in glycolysis, TCA cycle and oxidative phosphorylation. In addition, it also mediates calcium homeostasis, genetic expression, protection against oxidative stress, and programmed cell death (Ziegler, 2000). Under pathological conditions, such as cerebral ischemia, oxidative stress, hypoglycemia, or excitotoxicity, poly (ADP-ribose) polymerase-1 (PARP-1) is the most efficient depleting enzyme for NAD (Narasimhan et al., 2003; Kosenko et al., 2004). In this case, PARP becomes extremely active because it can promote the repair of damaged DNA. Activated PARP-1 hydrolyzes NAD, which causes a large decrease in intracellular NAD, especially under conditions of metabolic stress. At the same time, the reduction of intracellular ATP also limits the production of NAD. When the concentration of NAD is less than 1 mM or 0.1 mM, ATP production is affected, resulting in a vicious circle that eventually leads to permanent metabolic failure and necrotic cell apoptosis. Specific PARP-1 inhibitors DPQ or cyclosporine A have partial protection against the pathological process (Soane et al., 2007) (Table 1).

Mitochondrial dynamics are closely linked to ischemic brain injury. Severe brain ischemia caused mitochondrial fragmentation and dendritic damage (Kislin et al., 2017). Proteins mediated mitochondrial fission and fusion such as Drp1, Opa1, and Mfn1/2 exhibited dynamic expression in MCAO mice (Kumari et al., 2012; Liu et al., 2012; Martorell-Riera et al., 2014; Owens et al., 2015). Drp1 and Mfn1/2 mediated mitochondrial fission is required for ischemic neuronal death (Barsoum et al., 2006; Peng et al., 2015). Consistent with these observations, reducing mitochondrial fission by using mdivi-1, an inhibitor for Drp-1, exhibited protective roles against OGD reperfusion injury and MCAO through suppressing the ROS production and decreasing the expression of cytochrome c (Zhang N. et al., 2013; Wang et al., 2014). However, another study observed mdivi-1 exacerbated brain injury in a mouse model of transient focal ischemia (Zuo et al., 2014).

Upregulation of autophagy/mitophagy has been observed in the brains of rats subjected to permanent middle cerebral artery occlusion (MCAO). The upregulated mitophagy seems neural protective since inhibition of autophagy using 3-methyladenine (3-MA) exacerbated brain injury (Zuo et al., 2014). However, another two studies observed converse results that inhibition of autophagy by using either 3-MA or knocking-down of ATG7 exhibited neuroprotective in ischemia-induced neural injury (Zhang X. et al., 2013; Zuo et al., 2014) Enhancing autophagy with rapamycin attenuated mitochondrial dysfunction and partly reversed the deleterious effects of 3-MA-treated neurons subjected to oxygen-glucose deprivation reperfusion (Li et al., 2014). However, it is worth noting that excessive induction of mitophagy leads to cell death in neonatal stroke (Shi et al., 2014). Thus, it seems that mitophagy is a double-edged sword that can be protective or destructive after experimental stroke.

## Mitochondrial Dysfunction in Parkinson’s Disease

Parkinson’s disease (PD) is the second common neurodegenerative disease all over the world which demonstrates motor symptoms including myotonia, rest tremor, hypokinesia, and abnormal postures (Zeng et al., 2018) accompanied by non-motor symptoms such as anxiety, depression, constipation, frequent micturition, RBD and cognitive disorders (Marsili et al., 2018). Continuous loss of dopaminergic (DA) neurons and accumulation of α-synuclein (α-Syn) are regarded as the features of PD pathology (Bridi and Hirth, 2018; Song and Xie, 2018). Mitochondrial dysfunction plays essential roles in PD pathogenesis (Pozo Devoto and Falzone, 2017). One of evidence comes from the facts that treatment with toxin 1-methyl-4-phenyl-1, 2, 3, 6-tetrahydropyridine (MPTP), rotenone, trichloroethylene, yridaben, and other inhibitors of mitochondrial complex I reproduce the parkinsonian features including dopaminergic neurodegeneration in rodents and primates (Blesa et al., 2012). These toxins cause defects in the activity of the mitochondrial electron transport complex, mitochondrial movements and an increase in mitochondrial permeability transition and generation of ROS. Mutations in DJ-1, a mitochondrial peroxiredoxin-like peroxidase that plays a role in scavenging mitochondrial ROS, cause an autosomal recessive form of PD (Bonifati et al., 2003; Ammal Kaidery and Thomas, 2018), indicating a contribution of ROS in PD pathogenesis.

Accumulation of α-Syn in the brain is a noteworthy pathological feature in PD. Most of α-Syn localize at the cytoplasm, while a small fraction of α-Syn is present in mitochondria. α-Syn regulates the mitochondrial morphology through acting directly on the fusion-fission process by changing the membrane curvature (Kamp et al., 2010; Pozo Devoto and Falzone, 2017; Pozo Devoto et al., 2017). Distinct pathogenic variants that contain a single amino acid substitution in the N-terminal domain display different affinities for membranes, thus exhibiting distinct efficacy in inducing mitochondrial fragmentation: A53T mutations shows the most notable fragmentation effect, followed by mild fragmentation is observed by overexpression of WT α-Syn. Whereas almost no fragmentation is observed upon overexpression of the A30P variant (Nakamura et al., 2011; Butler et al., 2012; Gui et al., 2012; Pozo Devoto and Falzone, 2017; Pozo Devoto et al., 2017). However, some studies have reported that A30P α-Syn induces similar or even greater fragmentation defects to A53T (Kamp et al., 2010; Guardia-Laguarta et al., 2014). Striatal dopamine release deficits were restored after inhibition of Drp1 in animal models (Rappold et al., 2014), suggesting that the excessive fission contributes to the pathophysiology of PD. However, complete deletion of Drp1 in mice models leads to slower weight gain, parkinsonism, and loss of DA neuron in caudate–putamen (CPu) and nucleus accumbens (NAc) areas (Berthet et al., 2014), suggesting that the disruption of fission could also be a suspected factor in the pathogenesis of PD.

In addition to directly regulate the fusion-fission process of mitochondria, α-Syn also regulates mitochondrial transport. Overexpression of α-Syn leads to deficits of mitochondrial transport prior to axonal degeneration (O’Donnell et al., 2014; Pozo Devoto et al., 2017), which may be associated with impaired kinesin function and direct modulation of the fusion-fission dynamics (Pozo Devoto and Falzone, 2017).

Although there is still a lack of evidence as to whether α-Syn deregulates mitophagy in PD, studies have observed that α-Syn overexpression caused compromised autophagy activity, which was evidenced by increased levels of p62, decreased levels of LC3 and the number of LC3-II-positive vesicles (Winslow et al., 2010). α-Syn overexpression also leads to the mislocalization of autophagy related protein 9 (ATG9) via inhibiting Rab1 GTPase protein (Winslow et al., 2010). Moreover, mutant α-Syn, but not WT α-Syn, causes defects in the chaperon-mediated autophagy (CMA) pathway by binding to the LAMP2 transporter in the lysosomal membrane (Cuervo et al., 2004; Alvarez-Erviti et al., 2010). These evidences mentioned above hint that α-Syn accumulation or/and mutations participate in the pathogenesis of PD through regulating mitochondrial dynamics and mitophagy.

Other mutational genes coding Parkin, PINK1 (PTEN Induced Putative Kinase 1), LRRK2 (Leucine-rich repeat kinase 2), which are associated genetically with PD, have also been reported to alter mitochondrial dynamics. Mutations in Parkin and PINK1 are the common causes of autosomal recessive PD (Corti et al., 2011). These two genes operate together in a common genetic pathway. Recent studies show abnormality in either of them could cause mitochondrial dysfunction from unusual mitochondrial biogenesis, dynamics and mitophagy. For example, the reduced Complex I activity and impaired ATP production has been found in Parkin or/and PINK1 null PD patients or models, which may be resulted from impaired mitochondrial biogenesis (Muftuoglu et al., 2004; Morais et al., 2009). Supplementation of PINK1 restores PINK1-defect model fly’s ATP production and strength of flight muscles (Vos et al., 2012). Parkin exhibits protective function in maintaining mitochondrial genome integrity from mitochondrial DNA repair via an interaction with TFAM (Rothfuss et al., 2009). PARIS (Parkin Interacting Substrate, ZNF746), a strong pathogenic Parkin substrate, accumulates in familial PD with Parkin mutations, sporadic PD, Parkin knockout mice and MPTP- induced mice (Shin et al., 2011). PARIS upregulation is required for DA neuron degeneration caused by Parkin deficiency through repressing transcription of peroxisome proliferator-activated receptor gamma (PPARγ) coactivator-1α (PGC-1α) (Dawson and Dawson, 2014), a master regulator of mitochondrial biogenesis (Zhu et al., 2013). In this context, it is worth noting that PGC-1α levels are decreased in the SNc of PD patients (Shin et al., 2011). Parkin mediates mitophagy downstream of PINK1. PINK1 can act as a molecular sensor of damaged mitochondria. Parkin is an E3 ubiquitin ligase, which ubiquitylates numerous OMM proteins, which in turn recruits other proteins to mitochondria to initiate mitophagy. A recent study has observed that PINK1- and Parkin-mediated mitophagy restrains innate immunity through regulating STING levels (Sliter et al., 2018), highlighting mitochondrial dysfunction leads to neurodegeneration through neuroinflammation. In this context, it is worth noting that mitochondrial antigen presentation (MitAP), in which mitochondria-derived vesicles are targeted to endolysosomes for processing and presentation by MHC class molecules (MHC-1), is antagonized by Parkin and PINK1. MHC-1 are upregulated in DA neurons by inflammatory conditions. The absence of Parkin and PINK1 in familial PD promotes MitAP, results to the development of mitochondrial antigen-reactive CD8+ T cells, which might cross the BBB and bind to mitochondrial peptide: MHC-I complex, leading to the death of DA neurons in PD (Matheoud et al., 2016). PINK1/Parkin-mediated mitophagy can be attenuated by S-Nitrosylation of PINK1 (Oh et al., 2017), indicating a novel mechanism underlying that nitrosative stress damages mitochondria. Parkin and PINK1 also take part in the process of mitochondrial fusion. PINK1 phosphorylates Mfn, which tethers separated mitochondria together to induce fusion on the OMM, where Mfn is degraded by Parkin (Jin and Youle, 2012).

The LRRK2 (G2019S) mutation is another common cause of PD. LRRK2 regulates mitophagy by targeting RHOT1, an OMM protein, which anchors the microtubule motors to mitochondria. In both wild-type neurons and neurons differentiated from iPSC of human fibroblast, LRRK2 is recruited to RHOT-1 after mitochondrial depolarization or damage, and then removing RHOT-1 from OMM through promoting degradation by proteasomes. Through this way, LRRK2 leads to both anterograde and retrograde mitochondrial motility stop and subsequent mitophagy. While in mutant LRRK2 (such as LRRK2^G2019S^) cells, this phenomenon would be delayed, thus resulting in accumulation of depolarized or damaged mitochondria in cells, as well as higher oxidative stress (Hsieh et al., 2016). Consistently, knock-down RHOT1 in a drosophila model carrying human LRRK2^G2019S^ restores its motor disabilities and reduced DA neuronal numbers (Wang, 2017), implying that partial inhibition of RHOT1 may remit PD symptoms.

Given the potential role of mitochondrial dysfunction in PD pathogenesis, some therapeutics targeting mitochondria have been investigated. Selegiline has shown beneficial effect and significantly delaying the time of using levodopa through inhibiting monoamine oxidase-B (MAO-B) and antioxidant (Koller et al., 1993). Rasagiline, a new MAO-B inhibitor, enables to change the Unified Parkinson Disease Rating Scale (UPDRS) modestly (Smith et al., 2015). *N*-acetylcysteine (NAC) has been reported to improved UPDRS moderately (Monti et al., 2016) through protecting cells from mitochondrial dysfunction and from enhanced oxidative stress (Chandramani Shivalingappa et al., 2012). While other agents such as mitoquinone (mitoQ) (Snow et al., 2010; Liu and Wang, 2014; Xi et al., 2018), ubiquinone (Koller et al., 1993), creatine (Kieburtz et al., 2015) are still under investigation or demonstrated as no improvement in UPDRS (Table 1).

## Mitochondrial Dysfunction in Alzheimer’s Disease

Alzheimer’s disease (AD) is a neurodegenerative disease, which exhibits dementia syndrome characterized by learning and memory loss. AD is characterized with two pathological markers: amyloid-β (Aβ) plaques formed by accumulation of Aβ and neurofibrillary tangles (NFTs) consisted of hyperphosphorylated tau (Sun et al., 2018). In addition to these two pathological markers, mitochondrial dysfunction has been taken as another feature in both the familial and sporadic AD (Swerdlow et al., 2014). AD patients brain exhibit changes in the mitochondrial mass, enzymes and mtDNA, which are accompanied with altered morphology of mitochondria such as disruption of mitochondrial cristae, intra-mitochondrial accumulation of osmiophilic materials and changes in mitochondrial sizes (Silva et al., 2012). Neurons in the brains of AD patients exhibit less glucose uptake as revealed by the PET scan, suggesting enzyme activities that involved in mitochondrial oxidative phosphorylation and the tricarboxylic acid cycle (TCA) are reduced (Kapogiannis and Mattson, 2011). Extensive oxidative stress has been observed in the early stage of AD brains (Nunomura et al., 2001; Zhao and Zhao, 2013). The levels of oxidative markers are directly correlated with the severity of cognitive impairment as well as the symptomatic progression from mild cognitive impairment to AD (Ansari and Scheff, 2010), further suggesting a close link of mitochondrial dysfunction to AD pathology.

The mitochondrial dysfunction even occurs prior to the accumulation of Aβ plaques in the brains of AD mouse models. Thus, Swerdlow et al. (2014), has proposed the “mitochondrial cascade hypothesis” that mitochondrial dysfunction is taken as a trigger of AD pathogenesis. Consistent with this hypothesis, mitochondrial dysfunction pushes APP processing toward Aβ production (Gabuzda et al., 1994; Gasparini et al., 1997; Webster et al., 1998), enhances tau phosphorylation (Blass et al., 1990; Szabados et al., 2004), makes cells more susceptible to either Aβ- or tau-induced toxicity (Zhao and Zhao, 2013), eventually leading to neurodegeneration. One of the mechanisms underlying the above effects of mitochondrial dysfunction is oxidative stress. The extensive oxidative damages in AD brains result in oxidative modification of proteins and lipids, eventually leading to neuronal dysfunction. Additionally, the key enzymes for the generation of Aβ including BACE1 (the β-secretase in the brain) and PS1 (the core component of γ-secretase) are upregulated by oxidative stress (Tamagno et al., 2002; Oda et al., 2010). Oxidative stress also involved in Aβ- or tau- induced toxicity. Consistently, antioxidants such as curcumin and mitochondrial-targeted antioxidant mitoQ can attenuate the pathology of AD in model mice (Mecocci and Polidori, 2012; Zhao and Zhao, 2013). Some antioxidants are even under clinical trials in the therapy of MCI and AD (Mecocci and Polidori, 2012).

**FIGURE 3:**
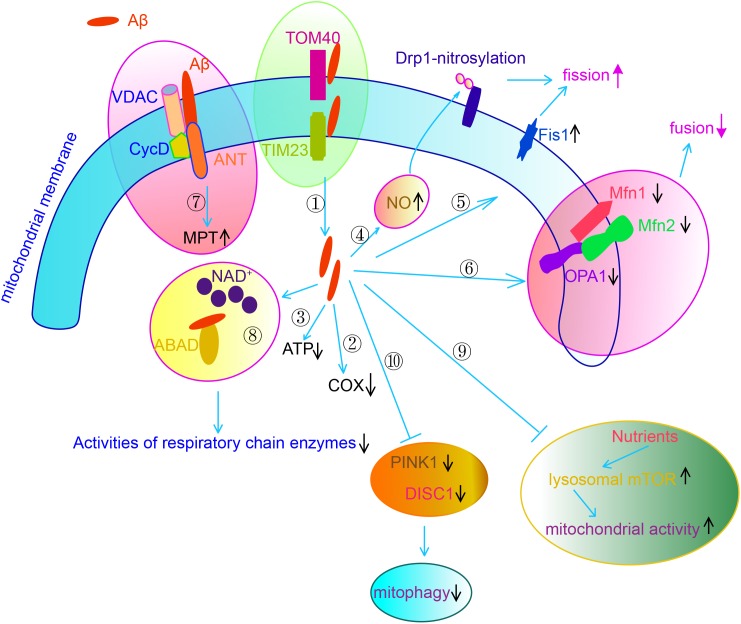
Aβ causes mitochondrial dysfunction via multiple mechanisms. 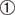 Aβ is imported to the mitochondria through interacting with TOM40 and TIM20, eventually localizing cross mitochondrial membrane. 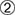 Aβ decreases COX activity. 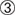 Aβ decreases ATP generation via binding to ATP synthase subunit α. 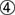 Aβ increases NO generation, which results in S-nitrosylation of DLP. 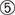 Aβ increases the expression of Fis1. Through 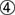 and 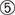, Aβ enhances mitochondrial fission. 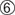 Aβ decreases the expression of Mfn1/2 and OPA1, through which Aβ suppresses mitochondrial fusion. 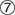 Aβ regulates mitochondrial permeability transition (MPT) via forming an interacting complex with ANT, cyclophilin D (Cyc D) and VADC, the core components of MPTP. 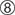 Aβ interacts with ABAD, preventing NAD+ to ABAD, thereby changing MTP and decreasing the activities of RC. 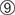 Aβ suppresses nutrients induced mitochondrial activity through regulation mTOR activity. 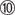 Aβ impairs mitophagy through downregulation of DISC1 and PINK1.

It is worth noting that accumulated Aβ and hyperphosphorylated tau also contribute to mitochondrial dysfunction in AD brains (Lu et al., 2018). Extracellular Aβ is internalized and imported to the mitochondria via interacting with TOM40 and TIM20, eventually localizing cross mitochondrial membrane (Hansson Petersen et al., 2008). Aβ interacts with adenine nucleotide translocase (ANT), cyclophilin D and voltage-dependent anion channel (VDAC), the key proteins in MPTP. Aβ also interacts with mitochondrial protein Aβ-binding ethanol dehydrogenase (ABAD), preventing the binding of NAD+ to ABAD, thereby changing MTP and decreasing the activities of RC enzymes (Lustbader et al., 2004). Aβ binds to ATP synthase subunit α and regulates the generation of ATP (Schmidt et al., 2008). Aβ accumulation also results in abnormalities of mitochondrial dynamics. APP decreases mitochondrial fusion/fission proteins such a as Drp1, OPA1, Mfn1 and Mfn2, while increases Fis1 significantly in AD neurons (Wang et al., 2008, 2009) through overproduction of Aβ (Wang et al., 2008). Another study observes that Aβ enhances mitochondrial fission via increasing generation of NO, which promotes S-nitrosylation of Drp1. Block nitrosylation of Drp1 through mutating cysteine abrogate Aβ-induced mitochondrial dysfunction and suppressed synaptic plasticity (Cho et al., 2009). Nutrients such as insulin or amino acids stimulates mitochondrial activity and regulates mitochondrial DNA synthesis in neurons by activating lysosomal mechanistic target of rapamycin complex 1 (mTORC1). However, Aβ oligomer suppresses such nutrient-induced mitochondrial activity through activating mTORC1 protein kinase activity at the plasma membrane but not at lysosomes in a way dependent on tau (Norambuena et al., 2018). Defective mitophagy also contributes to the accumulated damaged mitochondria in AD neurons. Both DISC1, a novel mitophagy receptor, and PINK1, which is a key regulator in mitophagy, exhibit reduced levels in the brains of transgenic mice. Overexpression of either DISC1 or PINK1 rescues mitochondrial dysfunction, cognitive, and synaptic deficits and Aβ accumulation in AD transgenic mice (Deng et al., 2016; Du et al., 2017; Wang et al., 2018) (Figure 3). Another recent study indicates that impaired syntaphilin-mediated retrograde axonal transport of mitochondria is involved in neurodegeneration of AD neurons in a way independent on Parkin-mediated mitophagy (Lin et al., 2017).

Tau is a microtubule-binding protein mainly responsible for the organization of microtubules in neurons. In this regard, hyperphosphorylated tau causes disruption of preformed microtubules and mitochondrial perinuclear distribution (Zempel et al., 2010; Amadoro et al., 2014; Musiek and Holtzman, 2015). P301L transgenic mice which overexpress the mutant P301L human tau protein exhibited abnormal expression of metabolism-related proteins including mitochondrial respiratory chain complex components, antioxidant enzymes, and synaptic proteins that are associated with increased oxidative stress (David et al., 2005). Overexpression of mutant P301L human tau, rather than WT tau, in neuroblast cells induces complex I deficit, which is accompanied by decreased ATP levels and increased susceptibility to oxidative stress (Schulz et al., 2012). Overexpression of mutant P301L human tau also decreases fusion and fission rates concomitant with downregulation of OPA-1 and Drp1. A recent study indicates that overexpression of human P301L tau completely inhibit mitophagy by impairing Parkin recruitment to defective mitochondria by sequestering it in the cytosol (Cummins et al., 2018). These lines of evidence highlight a role of hyperphosphorylated tau in regulation of mitochondrial dynamics (Quintanilla et al., 2014). Hyperphosphorylated tau also interacts with VDAC1, through which hyperphosphorylated tau blocks the mitochondrial pores (Manczak and Reddy, 2012).

Therefore, a vicious cycle also exists between mitochondria dysfunction and Aβ- or tau-pathology. Accumulation of Aβ or hyperphosphorylated tau causes mitochondrial dysfunction, which in turn execrates Aβ accumulation and tau hyperphosphorylation. The latter cause further damages on mitochondria through multiple mechanisms (Lu et al., 2018). Thus, various strategies that enhancing mitochondrial homeostasis have been used to treat AD in both animal models and clinical trials. Antioxidants such as CoQ10 and MitoQ show beneficial efficacy in AD transgenic mice (McManus et al., 2011; Muthukumaran et al., 2018). Idebenone, an analog of CoQ10, showed benefit effect in AD patients (Senin et al., 1992; Weyer et al., 1997). Supplementation with NAD+ precursors like nicotinamide riboside (NR) and nicotinamide mononucleotide (NMN) ameliorates cognitive deficits, synaptic plasticity, phosphorylation of tau in AD model mice (Wang et al., 2016; Hou et al., 2018). Amelioration of mitochondrial dynamic deficits by the treatment of mitochondrial division inhibitor 1 (mdivi-1), a mitochondrial fission inhibitor, attenuates cognitive and synaptic deficits and Aβ accumulation in AD transgenic mice (Baek et al., 2017; Wang et al., 2017). Consistent with the contribution of compromised mitophagy in AD pathogenesis, compounds enhancing autophagy/mitophagy attenuate AD pathology in AD model mice. For example, both rapamycin, which induces autophagy/mitophagy through suppressing mTOR, and trehalose, which induces mTOR-independent autophagy/mitophagy, exhibit benefit in AD model mice (Spilman et al., 2010; Caccamo et al., 2013; Du et al., 2013; Portbury et al., 2017). 2, 4- Dinitrophenol (DNP), one of mitochondrial uncoupling agents, which can induce autophagy, exhibits protective functions in APP/PS1 transgenic mice (Geisler et al., 2017) (Table 1).

## Outlook

Mitochondria play essential roles in various cellular functions. Mitochondrial dysfunction is closely linked to the pathogenic mechanisms of the acute or chronic neural injury in diseases. Dysregulation of mitochondrial biosynthesis, fission, fusion, and degradation are implicated as the potential mechanisms in these various neural injuries. Despite that differences in mechanisms underlying mitochondrial dysfunction in ischemia, PD and AD, enhancing mitochondrial homeostasis exhibits therapeutic effects in pre-clinical studies. Though few candidate drugs targeting mitochondria show efficacy in clinical trials, present knowledge of mitochondrial abnormalities in these diseases may still be helpful for us to make therapeutic schedules to slow down and even cure different kinds of neural injury by correcting the mitochondrial dysfunction mentioned above. Though challenges lie ahead, we are still full of confidence that we can overcome difficulties and eventually find the way out to cope with neural injury relating to dysfunctional mitochondria in the near future.

## Author Contributions

X-YZ, M-HL, D-JY, and W-LL wrote the first draft and revised it. D-EX, P-PY, M-HL, and C-XY prepared the literature. W-LJ and HC drew the figures. JT, Y-YX, and SL helped to revise the manuscript. Q-HM designed and revised the manuscript.

## Conflict of Interest Statement

The authors declare that the research was conducted in the absence of any commercial or financial relationships that could be construed as a potential conflict of interest.
